# Modelling and Calibration Technique of Laser Triangulation Sensors for Integration in Robot Arms and Articulated Arm Coordinate Measuring Machines

**DOI:** 10.3390/s90907374

**Published:** 2009-09-11

**Authors:** Jorge Santolaria, David Guillomía, Carlos Cajal, José A. Albajez, Juan J. Aguilar

**Affiliations:** Departamento de Ingeniería de Diseño y Fabricación, Centro Politécnico Superior, Universidad de Zaragoza, c/ María de Luna, 3, 50018 Zaragoza, Spain; E-Mails: daguisan@unizar.es (D.G.); ccajal@unizar.es (C.C.); jalbajez@unizar.es (J.S.S.); jaguilar@unizar.es (J.J.A.)

**Keywords:** laser triangulation sensor, articulated arm coordinate measuring machine, extrinsic and intrinsic calibration, non contact measurement, digitalization

## Abstract

A technique for intrinsic and extrinsic calibration of a laser triangulation sensor (LTS) integrated in an articulated arm coordinate measuring machine (AACMM) is presented in this paper. After applying a novel approach to the AACMM kinematic parameter identification problem, by means of a single calibration gauge object, a one-step calibration method to obtain both intrinsic—laser plane, CCD sensor and camera geometry—and extrinsic parameters related to the AACMM main frame has been developed. This allows the integration of LTS and AACMM mathematical models without the need of additional optimization methods after the prior sensor calibration, usually done in a coordinate measuring machine (CMM) before the assembly of the sensor in the arm. The experimental tests results for accuracy and repeatability show the suitable performance of this technique, resulting in a reliable, quick and friendly calibration method for the AACMM final user. The presented method is also valid for sensor integration in robot arms and CMMs.

## 1. Introduction

The progressive spread of reverse engineering and digitalization in metrology and quality control tasks has increased sensor integration needs in instruments traditionally used for dimensional metrology. The latest improvements in equipment accuracy have resulted in metrology instruments capable of obtaining quick and accurate measurements approaching those of conventional coordinate measuring machines under certain circumstances. LTSs, able to obtain 3D coordinates from the projection of a laser line onto the surface to be measured, are based on the triangulation principle and are mainly composed of a camera (CCD or APS and lens) and a laser diode with a cylindrical lens capable of projecting a plane. This way, it is possible to reconstruct *X*,*Y*,*Z* coordinates corresponding to the laser line points by combining information provided by the laser plane intersection with the surface to be measured and the camera perspective transformation matrix obtained during the sensor calibration.

The rapid integration of this type of 3D sensor in metrology equipment over recent years has been accompanied by a lack of standardization regarding their calibration procedures. For this reason different manufacturers have developed their own calibration procedures. However, these procedures do not reliably guarantee the accuracy of structured light optical measurement systems because they do not establish general evaluation procedures for the complete systems, due to the large number of parameters influencing the final system error. In particular, LTSs are nowadays the most commonly used non-contact sensors in traditional dimensional metrology equipment such as CMMs or AACMMs. This is due to their versatility and the fact that they are one of the most accurate structured light contactless measurement sensors, providing suitable accuracy values for most reverse engineering applications although, in general, these are not sufficient for metrological inspection tasks. AACMM applications with integrated laser sensors are, nowadays, mainly focused on the automotive, aeronautics and moulds sectors, and applications related to heritage conservation and general measurements of industrial components [1].

The difficulty of mathematically characterizing the influence of these parameters on the error in a general way for any LTS has traditionally prevented the development of calibration and later correction methods. Previous works [2] have tried to characterize the error mechanisms for a commercial LTS evidencing that, under optimal measurement conditions, the repeatability obtained for characteristic parameters measured from certain geometric primitives is better than 10 μm. On the other hand, the accuracy obtained with a CMM-mounted LST (capable of obtaining 60,000 pts/sec) when measuring gauge objects or compared with those of a CMM, ranges from tens of micrometers up to 0.5 mm. According to the above-mentioned studies, low values of repeatability indicate that it is necessary to establish effective error correction models to take advantage of the metrological characteristics of these kinds of devices. In recent years a substantial bibliography has appeared in the field of sensors based on image capture and structured light projection. In particular, concerning LTSs, the great majority of studies [3–6] have been focused on the proposal of mathematical models and calibration procedures for different types of sensor configuration and independent digitalization systems, analyzing in some cases in detail the influence on the final accuracy of specific error mechanisms [7,8]. Once the implemented mathematical models have been validated, based on the developments made in this field in the 1980s, the geometric configuration parameters of sensors are of extreme importance, as much as the digitalization conditions. The fact of not implementing error correction methods for each of the geometry and capture characteristic parameters error mechanisms makes it very difficult to increase sensor accuracy through mathematical models. At present, it is usual for commercial digitalization equipments to implement error correction mechanisms depending on the color of the piece to be digitalized. In addition, error correction methods based on lighting conditions and combinations of lens wavelength and electronic filters are used depending on the application.

Another conclusion reached by several authors [9] is that the main influence on the error of a LTS is the procedure establishing the relationship between the sensor frame and the its global support frame. Alternatively, the LTS-AACMM system calibration is different from the calibration of the sensor itself. This calibration, called intrinsic calibration, aims in this case to obtain the laser plane equation and to define the sensor reference frame, as well as to fix the relationship between screen coordinates *u*,*v* and *X*,*Y*,*Z* coordinates in the sensor frame. In addition, depending on the calibration model chosen, it is possible to describe the influence of other parameters, such as lens distortion. On the other hand, extrinsic calibration obtains the relationship between the sensor frame, defined during its intrinsic calibration, and the global reference frame of the sensor support for digitalization (CMM, AACMM, Robot,…), in which the digitized points will be obtained. As mentioned above, previous works have studied the intrinsic calibration of these types of sensors and the influence of the calibration process on the final error, as well as the development of optimization procedures for intrinsic parameters. Moreover, several authors have studied ways to solve the intrinsic and extrinsic calibration simultaneously [10] by means of techniques difficult to apply outside the laboratory.

Several possible assembly configurations using LTSs can be found nowadays in industry. Some use a CMM, a robot or an AACMM and others are assembled on specific high precision positioning systems or static structures under which the geometries to be digitized are displaced. The way to determine the relationship between the fixed frame (LTS or support) and the moving one will be of great influence on the final accuracy of the system.

Sensor manufacturers usually carry out the extrinsic calibration using a reference system on the sensor itself, making it necessary to subsequently transfer this extrinsic calibration to the LTS support. Several works have tried to solve this problem by scanning reference spheres in multiple spatial positions or by scanning the same sphere from three different sensor scanning paths in the same axis with a predefined offset between scans. Recent studies [9,11–13] have successfully solved the extrinsic calibration problem of LTS mounted in CMMs or machine-tools (MT) by establishing conjugated point pairs in both reference systems, taking advantage of the CMM or MT ability to move alternatively on a single axis, or simultaneously calibrating intrinsic and extrinsic parameters.

**Figure 1.**
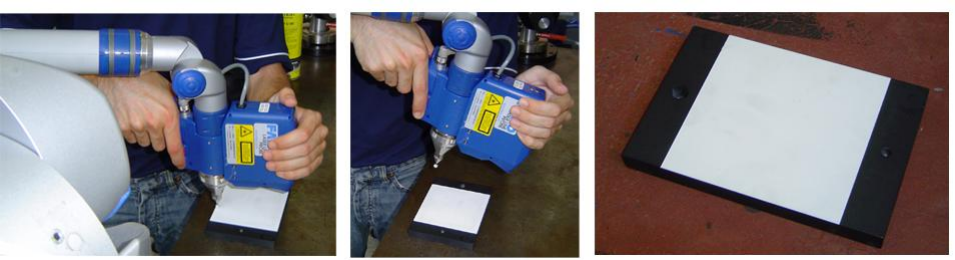
Contact and non contact measurement of a gauge plane to obtain an estimation of position and orientation of LTS coordinate system in AACMM last frame by optimization.

When the sensor is mounted on a manually operated AACMM, it is very difficult to apply these procedures without the aid of expensive instruments, because it is not possible to move the sensor only on a single axis of its reference system during the digitalization of points to obtain the conjugated pairs. Hence, it is necessary to find an alternative method of extrinsic calibration. This is the reason why LTS manufacturers carry out LTS or LTS-contact probe sets intrinsic and extrinsic calibration prior to mounting the sensor on a CMM. After that, if the set is calibrated, the extrinsic calibration is reduced to obtain, by the above-mentioned procedures, the geometric relationship between the LTS reference system and the contact probe reference system. Later, the complete set is mounted in an AACMM, integrating the sensor in its mathematical model through its well-known relation with the contact probe. If only the LTS has been calibrated, the usual technique for further integration of LTSs in AACMMs consists of mounting the sensor and digitizing a reference geometric primitive, usually a plane, from some spatial orientations. Later, a contact measurement of the same primitive is carried out with the AACMM (Figure 1). This way, it is possible to approximate initial values of the extrinsic parameters, involving rotation and translation, and to obtain the initial matrix that transforms coordinates in the sensor reference system to the last reference system of the arm. With this initial matrix and the nominal values of the contact measured primitive, it is possible to establish an optimization procedure that minimizes the digitized points error in the global coordinate system of the arm, varying successively the value of the extrinsic parameters. Finally, after the optimization procedure, the matrix that minimizes the digitalization error at the chosen capture positions is obtained.

This method is common in combined AACMM-LTS commercial systems in which the manufacturer of the LTS performs the intrinsic calibration of the sensor and defines, by means of local extrinsic calibration, its reference system. The later integration of the sensor in the AACMM is carried out by the previously described approximate determination procedure of the above-mentioned matrix. During this procedure, the capture of points of the digitized primitive requires manual displacements of the measurement arm that gather the influence of errors due to kinematic parameters and of dynamic errors, which are generally dependant on the position of the joints at the moment of digitalization. These errors are later reduced by the optimization procedure to obtain the final extrinsic parameters that will transform coordinates in the sensor coordinate system to the global arm coordinate system for any arm position and orientation. Therefore, a transformation matrix is obtained that is highly dependant on the digitized zone of the gauge primitive and only suitable for digitalization trajectories similar to those used during the data capture.

In this work, the mathematical modelling of a commercial LTS is presented first, followed by the complete procedure of sensor intrinsic and extrinsic calibration with the LTS already mounted in the AACMM. This method circumvents the use of approximated methods to determine extrinsic parameters, such as the digitalization of gauge objects prior to extrinsic calibration of the sensor, thus also avoiding the introduction of possible digitalization errors during scanning paths for extrinsic calibration. Moreover, the transformation matrix is obtained analytically in a single step. This is necessary in order to obtain 3D coordinates in the AACMM global coordinate system from the laser line points image coordinates in any capture position. Therefore, the time and the cost necessary to calibrate the whole equipment with current methods are reduced.

## 2. AACMM Kinematic Modelling and Parameter Identification

The kinematic model of the arm, the parameters considered, and the identification process used [14] are briefly explained here as the initial step in the sensor integration technique described here.

### 2.1. Kinematic Modelling

In the present work, the AACMM used is a six degrees-of-freedom (dof) Sterling series FARO arm with a typical 2-2-2 configuration and a-b-c-d-e-f deg rotation, in accordance with ASME B89.4.22-2004. Each of the six joints is characterized by the four geometrical parameters (distances *d_i_*, *a_i_* and angles *θ_i_*, *α_i_*) defined in the basic Denavit-Hartenberg (D–H) model [15] used for the AACMM studied. The D–H model uses these parameters to calculate the transformation of coordinates between successive reference systems linked to the arm joints. The homogeneous transformation matrix between frame i and i-1 depends on these four parameters:

(1) $${}^{i-1}\;A_{i}=T_{z,d}T_{z,\theta }T_{x,a}T_{x,\alpha }=\left[\begin{array}{cccc} {\text{cos}\;\theta }_{i} & -\text{cos}\;\alpha_{i}\;\text{sin}\;\theta_{i} & \text{sin}\;\alpha_{i}\;\text{sin}\;\theta_{i} & a_{i}\;\text{cos}\;\theta_{i}\\ \text{sin}\;\theta_{i} & \text{cos}\;\alpha_{i}\;\text{cos}\;\theta_{i} & -\text{sin}\;\alpha_{i}\;\text{cos}\;\theta_{i} & a_{i}\;\text{sin}\;\theta_{i}\\ 0 & \text{sin}\;\alpha_{i} & \text{cos}\;\alpha_{i} & d_{i}\\ 0 & 0 & 0 & 1 \end{array}\right]$$

**Figure 2.**
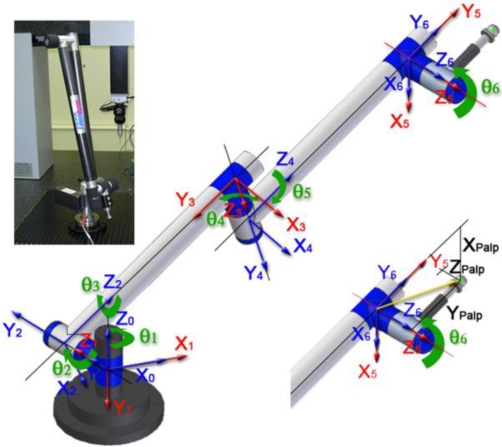
Model definition posture of FARO AACMM with D–H convention [14].

In Equation (1), the joint variable *θ_i_* of the model is related to the rotation reading provided by the encoder through Equation (2), where *θ_0i_* must also be identified from its nominal value defined for the initial position chosen for the model. This way, the arm model depends on a total number of 27 parameters to be identified. The AACMM used and its initial position for the model are shown in Figure 2.

(2) $$\theta_{i}=\theta_{\mathrm{iEnc}}-\theta_{0i}$$

By means of successive transformations of the coordinates, by pre-multiplying successively the transformation matrix for a given position between a frame and the previous one, it is possible to obtain the global transformation matrix of the arm, which gives the coordinates of the centre of the probe sphere with regards to the base of the AACMM.

(3) $$\begin{array}{ll} {}^{0}T_{6}={}^{0}A_{1}{}^{1}A_{2}{}^{2}A_{3}{}^{3}A_{4}{}^{4}A_{5}{}^{5}A_{6} & {\bar{X}}_{\mathrm{AACMM}}={}^{0}T_{6}{\bar{X}}_{\mathrm{Probe}} \end{array}$$

In this manner, considering 0 as the global fixed reference system of the base and 6 as the reference system moving with the rotation of the last joint (Figure 2), the desired homogeneous transformation and coordinates can be obtained by way of Equation (3).

### 2.2. Data Capture and Non-Linear Least Squares Identification Scheme

**Figure 3.**
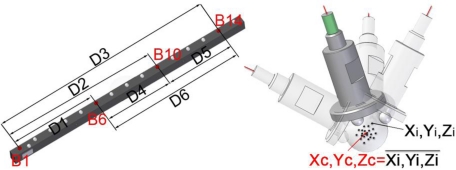
Balls measured and distances between sphere centres calculated [14].

All the calibration procedures, both for robotic arms and AACMMs, are based on the establishment of a system which materialises coordinates or nominal distances in the workspace, in order to capture points which allow the error to be evaluated and minimized. The number of identification and data capture methods for robotic arms contrasts with the scant bibliographical resources regarding capture methods for parameter identification in measurement arms, currently identical to those used in robot parameter identification techniques. The different nature of robot arms and AACMMs requires the development of strategies to obtain the results desired in each case. A continuous data capture method has been implemented [14], allowing the massive and quick capture of arm positions corresponding to several points of the workspace. To this end, a ball-bar gauge 1.5 m long was placed in seven positions within the workspace of the arm in order to cover the maximum number of possible AACMM positions, in order to subsequently extrapolate the results obtained throughout the volume to measurement positions not used in the identification process. The ball-bar comprises a carbon fibre profile and 15 ceramic spheres of 22 mm in diameter. Thus, calibrated distances are available between the centres with an uncertainty, in accordance with its calibration certificate, of (1 + 0.001·L) μm, with L in mm. A specific probe was designed capable of directly probing the centre of the gauge spheres instead of having to probe their surface points. Figure 3 shows the balls and distances considered, and also the self-centring passive probe.

Therefore, apart from characterising and optimising the behaviour of the arm with regard to error in distances, its capacity to repeat measurements of a same point is also tested and subsequently optimized. Hence, automatic arm position capture software has been developed, probing each considered sphere of the gauge and replicating the arm behaviour in the ASME B89.4.22-2004 single-point articulation performance test, but in this case including the positions captured in the optimization from the point of view of this repeatability. The objective function used in the Levenberg-Marquardt [16,17] based identification algorithm, presented in Equation (4), shows the influence of the arm behaviour with regard to volumetric accuracy and point repeatability, minimizing simultaneously the errors corresponding to both parameters:

(4) $$\phi =\sum_{i=1}^{r}\sum_{j,k=1}^{s}\left[{\left(D_{i_{\mathrm{jk}}}-D_{0jk}\right)}^{2}+{\left({2\sigma }_{\mathrm{Xij}}\right)}^{2}+{\left({2\sigma }_{\mathrm{Yij}}\right)}^{2}+{\left({2\sigma }_{\mathrm{Zij}}\right)}^{2}\right]$$

Where *D_i_jk__* represents the Euclidean distance between sphere j and sphere k of the gauge i location, with coordinates corresponding to the mean of the points captured for sphere j and sphere k, *D*_0*jk*_ the nominal distance materialized by the gauge and *σ_Xij_* the standard deviation in the x coordinate of the points captured for the sphere j in position i of the gauge. Analogous for y and z coordinates. In Equation (4), r = seven positions of the ball bar and s = four spheres (1, 6, 10 and 14) per bar position are considered. From the initial values of the parameters, obtained from their nominal value in the model definition position, Table 1 shows the AACMM kinematic model parameters finally identified. The error values obtained for the identified set of parameters and data captured are shown in Table 2. These contact measurement maximum error values have to be considered in the subsequent evaluation of the whole system.

## 3. Sensor Modelling

LTS modelling must establish the geometric relations necessary to obtain 3D coordinates, in the global coordinate system, of the points from the 2D CCD image corresponding to the line formed by the intersection of the laser plane and the surface to be digitized. The parameters to consider and to calibrate subsequently by means of the implemented method include intrinsic and extrinsic parameters of the camera and the laser plane equation.

### 3.1. Camera Modelling

**Figure 4.**
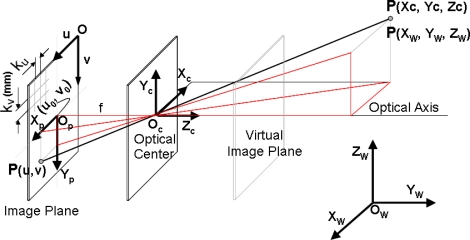
Perspective projection of pin-hole camera model without distortion [11].

The basic camera model, based on the perspective projection principle, obtains *u* coordinates of an image point as a non-linear function of the point in the 3D global coordinate system and the extrinsic and intrinsic parameters:

(5) u=P(X,θ)

where *u* = (*u*,*v*)*^T^* are the point coordinates in the 2D image coordinate system, *X* = (*x*,*y*,*z*)*^T^* the point coordinates in the sensor global coordinate system, and *θ* = (*θ_int_*,*θ_ext_*)^T^ a vector with the intrinsic and extrinsic camera parameters. Although there are diverse approaches to the consideration of these parameters, the basic intrinsic parameters make reference firstly to the geometry and optics of the camera, involving: (1) focal length *f*, (2) *u*_0_ and *v*_0_ coordinates of the principal point in pixels, (3) *k_i_* distortion parameters according to the distortion model chosen, and secondly to the CCD sensor geometry, involving the aspect ratio which determines the length *k_u_* and width *k_v_* in mm. of the sensor pixels. The extrinsic parameters determine the position and orientation of the camera in the LTS global coordinate system, expressed in any one of the possible formulations of transformations between coordinates systems. The perspective projection principle for camera modelling is shown in Figure 4.

From Figure 4, and assuming a virtual image plane at distance f in the positive Zc axis, the coordinates of an image point may be expressed by means of Equation (6).

(6) $$\begin{array}{l} u\left(\mathrm{px}\right)=X_{p}\left(\mathrm{mm}\right)k_{u}\left(\frac{\mathrm{px}}{\mathrm{mm}}\right)+u_{0}\left(\mathrm{px}\right)\\ v\left(\mathrm{px}\right)=Y_{p}\left(\mathrm{mm}\right)k_{v}\left(\frac{\mathrm{px}}{\mathrm{mm}}\right)+v_{0}\left(\mathrm{px}\right) \end{array}$$

From Equation (6), and operating with the relations in the pin-hole model, it is possible to obtain the well known expression shown in Equation (7), where *PTM*, known as the perspective transformation matrix, is a matrix whose terms are a linear combination of the considered intrinsic and extrinsic parameters, as shown in Equation (8):

(7) $$\left[\begin{array}{c} \mathrm{su}\\ \mathrm{sv}\\ s \end{array}\right]=\mathrm{PTM}\left[\begin{array}{c} X_{W}\\ Y_{W}\\ Z_{W}\\ 1 \end{array}\right]$$

(8) $$\mathrm{PTM}=\left[\begin{array}{cccc} \alpha_{u}r_{11}+u_{0}r_{31} & \alpha_{u}r_{12}+u_{0}r_{32} & \alpha_{u}r_{13}+u_{0}r_{33} & \alpha_{u}t_{x}+u_{0}t_{z}\\ \alpha_{v}r_{21}+v_{0}r_{31} & \alpha_{v}r_{22}+v_{0}r_{32} & \alpha_{v}r_{23}+v_{0}r_{33} & \alpha_{v}t_{y}+v_{0}t_{z}\\ r_{31} & r_{32} & r_{33} & t_{z} \end{array}\right]$$

Equation (7) allows us to obtain the 2D screen coordinates that correspond to a 3D point, the coordinates of which are known in the sensor global coordinate system. In this case, with the camera model proposed, the aim is to obtain global coordinates from the extracted information of the images and, therefore, from the screen coordinates. The *PTM* is a noninvertible matrix, which is why it is necessary to find resolution methods that do not imply the inversion of this matrix. Thus, it is possible to express Equation (7) in terms of unknown coefficients in order to subsequently propose the resolution algorithm that will determine the coefficients of the perspective transformation matrix, shown in Equation (9):

(9) $$\left[\begin{array}{c} \mathrm{su}\\ \mathrm{sv}\\ s \end{array}\right]=\left[\begin{array}{cccc} m_{11} & m_{12} & m_{13} & m_{14}\\ m_{21} & m_{22} & m_{23} & m_{24}\\ m_{31} & m_{32} & m_{33} & m_{34} \end{array}\right]\;\left[\begin{array}{c} X_{W}\\ Y_{W}\\ Z_{W}\\ 1 \end{array}\right]$$

Expressing Equation (9) in explicit form:

(10) $$s\cdot u=m_{11}\cdot X_{w}+m_{12}\cdot Y_{w}+m_{13}\cdot Z_{w}+m_{14}$$

(11) $$s\cdot v=m_{21}\cdot X_{w}+m_{22}\cdot Y_{w}+m_{23}\cdot Z_{w}+m_{24}$$

(12) $$s=m_{31}\cdot X_{w}+m_{32}\cdot Y_{w}+m_{33}\cdot Z_{w}+m_{34}$$

Only two of the three equations obtained are linearly independent. Thus, operating with these equations, it is possible to extract the linearly independent equations from (10)–(12)*u* and (11)–(12)*v*, obtaining the expressions in Equation (13):

(13) $$\left\{\begin{array}{l} m_{11}\cdot X_{w}+m_{12}\cdot Y_{w}+m_{13}\cdot Z_{w}-m_{31}\cdot u\cdot X_{w}-m_{32}\cdot u\cdot Y_{w}-m_{33}\cdot u\cdot Z_{w}-u\cdot m_{34}+m_{14}=0\\ m_{21}\cdot X_{w}+m_{22}\cdot Y_{w}+m_{23}\cdot Z_{w}-m_{31}\cdot u\cdot X_{w}-m_{32}\cdot v\cdot Y_{w}-m_{33}\cdot v\cdot Z_{w}-v\cdot m_{34}+m_{24}=0 \end{array}\right\}$$

Equation (13) represents the equation of a straight line in the space which connects the point in the 3D global reference system with the point in the image. In this manner, if a point in the global reference system is known, its corresponding screen coordinates can be reconstructed. Since the ultimate purpose of sensor modelling and calibration is to achieve the mechanism to obtain 3D coordinates from information of points in known image coordinates, Equation (13) defines a system of two equations with three variables. This is the reason why more information is necessary to obtain the required coordinates. Once the camera is modelled, its later calibration will provide the values of the *m_ij_* coefficients of the perspective transformation matrix.

### 3.2. Laser Plane Modelling

The aim of the LTS is to obtain the coordinates expressed in the 3D global coordinate system of the points identified in an image belonging to the laser plane, through its projection onto the piece to be digitized. Therefore, a point *M* identified in the image belonging to the intersection line with the surface to be digitized will have to fulfil the camera model equations. Besides, this point also belongs to the laser plane. Thus, the laser plane is modelled by the general equation of a plane expressed in the global coordinate system:

(14) $${\mathrm{cAX}}_{w}+{\mathrm{cBY}}_{w}\;+\;{\mathrm{cCZ}}_{w}+\mathrm{cD}=0$$

**Figure 5.**
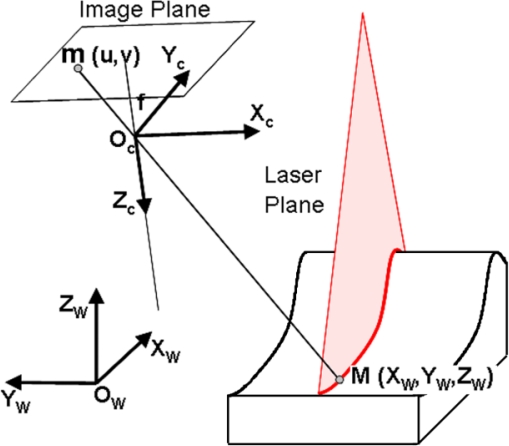
The global coordinates of a point *M* in the laser line image are computed from camera model and laser plane equation [11].

The laser plane contributes with the additional information necessary to complete the equation of the straight line of the camera model and to achieve a system of three equations with three variables for each identified point, so that their 3D global coordinates can be extracted from their 2D screen coordinates *u*, *v* (Figure 5).

## 4. Calibration Method

AACMM-LTS integration demands the determination of the geometric relationships between the LTS frame and the AACMM last joint frame or, in other words, the extrinsic parameters of the sensor once integrated in the arm. Thus, the extrinsic calibration procedure of a LTS mounted on an AACMM consists of determining the sensor frame origin coordinates and its direction related to the AACMM last joint frame, linking both mathematical models. Once these extrinsic parameters are determined, the laser line point coordinates are obtained in the LTS frame and, therefore, also with respect to the AACMM global frame in any arm pose.

Traditional integration methods are based on digitalization of gauge geometric primitives, generally planes or spheres. These methods start with the sensor already calibrated. Then, they perform multiple scanning paths over the gauge primitive without knowing the geometric relationship between the LTS frame and the last joint frame of the AACMM. By comparing the contact measurement of the gauge primitive, taken as nominal, and the least-squares one reconstructed from digitized points, it is possible to define a measurement error. The matrix that links the mathematical model of the sensor with the mathematical model of the AACMM is then obtained by an optimization procedure that minimizes the error mentioned changing the terms of such unknown matrix starting from an approximate initial value. Thus, the obtained matrix allows to subsequently expressing the coordinates of the digitized points in the AACMM global frame. The optimization methods used in these techniques are commonly based on the gradient method, so the success of the optimization procedure and its speed of convergence depend on the initial value considered for the matrix terms.

The calibration method presented in this section performs the intrinsic and extrinsic calibration of the sensor in a single step, so it is not necessary to have the LTS previously calibrated. Furthermore, it is based on the capture of an image of a gauge object in a single AACMM position, so the error influence of the arm due to the error made during the scan paths is avoided, absorbing only the measurement error in the contact measurement procedure of the gauge object and the error in the AACMM capture position of the image for calibration. Finally we obtain the transformation matrix between the LTS reference system and the last joint frame of the arm following an analytical scheme, thus avoiding optimization procedures. The result of these optimization procedures depends on the type and number of scanning paths because it adjusts the matrix terms to minimize the error with the captured data in each case. With the proposed method explained in this section, the digitization of a geometric primitive is also avoided.

On the other hand, there are many influences over the final accuracy of a LTS. The digitization of a geometric primitive using a manually operated instrument like an AACMM implies that it is not possible to maintain constant neither the distance from the sensor to the scanned surface nor the perpendicular orientation of the laser to the surface of the gauge primitive, affecting also the manual operation to the digitalization conditions such as scanning speed. The use of scanning paths in the traditional methods implies that the digitized points will be affected by these error sources. Thus, these errors will be subsequently absorbed by the least squares based calculation of the gauge geometric primitive with the captured points, and by the optimization procedure of the link matrix, affecting the final accuracy depending on the data and scans considered.

This section presents the required steps to perform the calibration method presented, which avoids data-dependent optimization procedures and the consideration of an initial value for the matrix terms, and also influences of the mentioned error mechanisms.

### 4.1. Calibration Points Location in AACMM Measurement Volume

**Figure 6.**
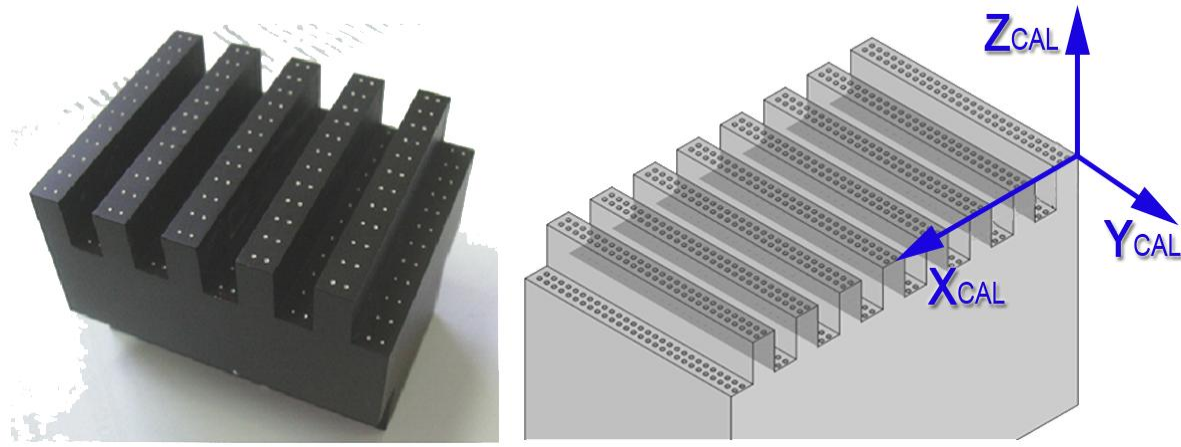
Gauge object with calibration points [11].

The target object used in this work is shown in Figure 6. It is a high precision gauge object that materializes points of well-known nominal coordinates in its local coordinate system, located in its upper left corner. It has points distributed in different planes that will allow the later calibration of the LTS camera and laser plane. The gauge object has a maximum flatness error of 0.003 mm and an average dot diameter of 0.799 mm, providing nominal coordinates of the points.

**Figure 7.**
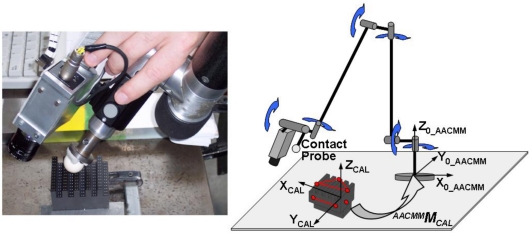
Gauge object contact measurement. Alignment of AACMM and calibration object coordinate systems.

The first step of the implemented integration procedure consists of the alignment of the AACMM reference system with the gauge object coordinate system. Once placed in a position accessible to the arm, the gauge object is measured by contact with the AACMM to align a reference frame attached to the calibration object and calculate a transformation matrix *^AACMM^M_CAL_* to know the calibration object points in the AACMM global frame (Figure 7). The accuracy obtained in the calculation of the points coordinates of the gauge object in the AACMM global reference system depends on the correct alignment of both reference systems during the contact measurement. Nine points are probed in each of the three planes that form the upper left corner of the gauge object, where its nominal reference system is located. Thus, it is possible to reconstruct the reference frame where the nominal coordinates of the points are expressed, taking the normal vector to the upper plane as the Z axis, the intersection of the upper and the longitudinal plane as X axis and the cross product as Y axis. From the coordinates of the vectors of the reference system it is possible to know the components of the homogeneous transformation matrix between the reference system reproduced on the gauge object and the global reference system of the AACMM. The accuracy of the final link matrix between the LTS and the last joint frame of the arm depends on the suitable alignment of these reference systems, so that AACMM repeatability and kinematic parameters induced errors in this contact measurement will be propagated to the final matrix obtained.

The *^AACMM^M_CAL_* is a 4 × 4 homogenous transformation matrix that transforms gauge object coordinates into AACMM global frame coordinates as shown in Equation (15):

(15) $${\left[\begin{array}{c} x_{i}\\ y_{i}\\ z_{i}\\ 1 \end{array}\right]}_{\mathrm{AACMM}}={}^{\mathrm{AACMM}}M_{\mathrm{CAL}}{\left[\begin{array}{c} x_{i}\\ y_{i}\\ z_{i}\\ 1 \end{array}\right]}_{\mathrm{CAL}}$$

### 4.2. LTS Calibration

In the current integration procedures based on error optimization over digitalized data, once the LTS intrinsic calibration has been done on a CMM, due to the point reconstruction process nature used in the LTS model, only points belonging to the captured laser line are known in the LTS frame when the LTS is linked to the arm. Thus, the coordinates of these points in the AACMM global frame cannot be obtained in this situation. In order to avoid approximate optimization procedures so as to determine the sensor position and orientation in AACMM coordinate system, it is necessary to do the LTS intrinsic calibration once it is already mounted onto the AACMM, when the camera gauge object point coordinates can be known in the LTS frame. LTS calibration implies the determination of the intrinsic and extrinsic parameters of the camera, and therefore the terms of the perspective transformation matrix of Equation (13) expressed in its coordinate system. The terms of the laser plane equation in this coordinate system, shown in Equation (14), are also obtained during LTS calibration.

**Figure 8.**
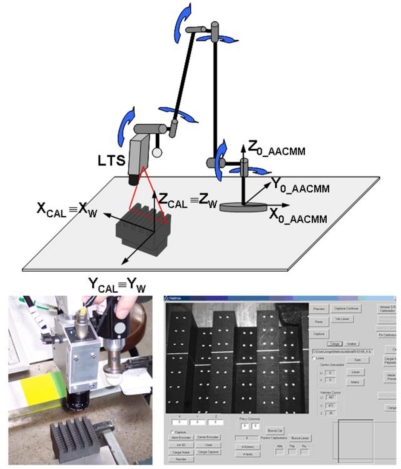
Image capture for LTS intrinsic calibration in AACMM calibration position.

Once the calibration object point coordinates in the AACMM global reference frame are known by means of Equation (15), an image of the calibration object is captured with the LTS in a single AACMM posture. This image, as can be appreciated in Figure 8, must contain the points of the gauge object and the laser line corresponding to the intersection of the LTS plane with the object.

From the captured image it is possible to determine the image coordinates *u*, *v* in pixels, corresponding to the centre of each one of the object points by means of centroid calculation. Since the perspective transformation matrix of Equation (9) has 12 unknown components and for each identified point there are two equations, Equation (13), at least six points of the gauge will be necessary to perform the sensor calibration.

The perspective transformation matrix being homogenous, the solution is modified by a scale factor, reason why the condition *m_34_* = 1 is imposed, considering that this term is not null since *t_z_* contains the term corresponding to the camera coordinate system translation to the LTS global coordinate system. It is possible to obtain the subsequent scale factor to be applied on the obtained matrix forcing the vector formed by the three first components of the last row of the matrix to be unitary. This scale factor will match with the translation *t_z_*. In these conditions it is possible to write in matrix form the equations obtained according to Equation (13) for each considered calibration point, obtaining a system of equations in the form of Equation (16):

(16) Am=0

where,

(17) $$A=\left[\begin{array}{cccccccccccc} X_{\mathrm{Wi}} & Y_{\mathrm{Wi}} & Z_{\mathrm{Wi}} & 1 & 0 & 0 & 0 & 0 & -u_{i}X_{\mathrm{Wi}} & -u_{i}Y_{\mathrm{Wi}} & -u_{i}Z_{\mathrm{Wi}} & -u_{i}\\ 0 & 0 & 0 & 0 & X_{\mathrm{Wi}} & Y_{\mathrm{Wi}} & Z_{\mathrm{Wi}} & 1 & -v_{i}X_{\mathrm{Wi}} & -v_{i}Y_{\mathrm{Wi}} & -v_{i}Z_{\mathrm{Wi}} & -v_{i}\\ X_{\mathrm{Wi}+1} & Y_{\mathrm{Wi}+1} & Z_{\mathrm{Wi}+1} & 1 & 0 & 0 & 0 & 0 & -u_{i+1}X_{\mathrm{Wi}+1} & -u_{i+1}Y_{\mathrm{Wi}+1} & -u_{i+1}Z_{\mathrm{Wi}+1} & -u_{i+1}\\ 0 & 0 & 0 & 0 & X_{\mathrm{Wi}+1} & Y_{\mathrm{Wi}+1} & Z_{\mathrm{Wi}+1} & 1 & -v_{i+1}X_{\mathrm{Wi}+1} & -v_{i+1}Y_{\mathrm{Wi}+1} & -v_{i+1}Z_{\mathrm{Wi}+1} & -v_{i+1}\\ \vdots & \vdots & \vdots & \vdots & \vdots & \vdots & \vdots & \vdots & \vdots & \vdots & \vdots & \vdots \\ X_{\mathrm{Wn}} & Y_{\mathrm{Wn}} & Z_{\mathrm{Wn}} & 1 & 0 & 0 & 0 & 0 & -u_{n}X_{\mathrm{Wn}} & -u_{n}Y_{\mathrm{Wn}} & -u_{n}Z_{\mathrm{Wn}} & -u_{n}\\ 0 & 0 & 0 & 0 & X_{\mathrm{Wn}} & Y_{\mathrm{Wn}} & Z_{\mathrm{Wn}} & 1 & -v_{n}X_{\mathrm{Wn}} & -v_{n}Y_{\mathrm{Wn}} & -v_{n}Z_{\mathrm{Wn}} & -v_{n} \end{array}\right]$$

and

(18) $$m={\left[\begin{array}{cccccccccccc} m_{11} & m_{12} & m_{13} & m_{14} & m_{21} & m_{22} & m_{23} & m_{24} & m_{31} & m_{32} & m_{33} & m_{34} \end{array}\right]}^{T}$$

Knowing the coordinate pairs *u*, *v* and *X_W_*, *Y_W_*, *Z_W_* corresponding to *n* = 42 calibration points in the captured image, it is possible to obtain the perspective transformation matrix. To do this, it is necessary to rearrange the system in Equation (16) and to write it in a non-homogenous form:

(19) A′m′=b

where *A’* is the matrix formed by the 11 first columns of *A*, *b* the column vector with the 12th column of *A* and *m’* a vector that contains all the elements of the perspective transformation matrix except *m_34_*. In these conditions it is possible to propose a least squares resolution scheme based on the pseudoinverse matrix, in the form of Equation (20), that will obtain the estimated values for the perspective transformation matrix coefficients included in the *m’* vector:

(20) $$m\prime ={\left({A\prime }^{T}A\prime \right)}^{-1}{A\prime }^{T}b$$

LTS calibration, in addition to giving the camera intrinsic parameters, defines the position and orientation of the sensor global coordinate system. In this way, by means of this calibration, the sensor global coordinate system is defined coincident with the gauge object local coordinate system (Figure 6), in which are known the 3D point coordinates.

On the other hand it is necessary to identify the screen coordinates of the laser line points in order to determine the equation that defines the laser plane in the coordinate system considered. The captured laser line has a greater width than a single pixel in the image, which is the reason why the identification of the laser line point is carried out by means of a gray level centroid estimation algorithm for each cross section of the line [18]. As much the width as the marking uniformity of the line have a direct influence on the final accuracy of the digitized points. This technique produces sub-pixel detection in the determination of *u*, *v* coordinates for the laser line points. The aim of using a crenellated gauge object is to have non coplanar calibration points that cover the complete final LTS measurement range in the camera optical axis direction, 10 mm in this particular case.

Once the laser line points screen coordinates have been detected, and considering that the *Z_W_* coordinate of the gauge object planes and therefore of the laser line points on these planes is known in the global coordinate system, it is possible to obtain the X_W_, Y_W_ coordinates of the identified points using Equation (13). After the camera calibration, the perspective transformation matrix coefficients are known, the reason why Equation (13) represents in this case a system of two equations with two variables for each identified point of the laser line. In this way, with the 3D coordinates of the laser line points known in the global coordinate system, it is possible to follow a least squares resolution scheme to determine the coefficients of Equation (14), assuming that every point in the laser line must fulfil the plane equation in addition to the camera model. The plane that best fits to the identified line points is then calibrated and well-known in the LTS global coordinate system.

Finally, in the subsequent LTS operation, the information provided by the laser plane complements Equation (13) and allows the reconstruction of the 3D global coordinate system by means of Equation (21) applied to each point, from the screen coordinates *u*, *v* after the identification of the points in the image:

(21) $$\left[\begin{array}{ccc} m_{11}-m_{31}\cdot u & m_{12}-m_{32}\cdot u & m_{13}-m_{33}\cdot u\\ m_{21}-m_{31}\cdot u & m_{22}-m_{32}\cdot v & m_{23}-m_{33}\cdot v\\ \mathrm{cA} & \mathrm{cB} & \mathrm{cC} \end{array}\right]\cdot \left[\begin{array}{c} X_{w}\\ Y_{w}\\ Z_{w} \end{array}\right]=\left[\begin{array}{c} -u+m_{14}\\ -v+m_{24}\\ \mathrm{cD} \end{array}\right]$$

It is necessary to note that the sensor calibration has been shown without considering distortion effects on the reconstructed points. These effects are very low in the modelled sensor because the capture distance to the surface only allows the capture of points in the range of ±5 mm around the central line of the captured image, where the distortion effects are minimum. In order to verify distortion effects on the reconstructed points, the calculation of the screen coordinates corresponding to the gauge object points after the calibration has been made, obtaining mean values of 0.224 pixels in maximum error for the *u* coordinate and 0.233 for the *v* coordinate in several calibration tests.

### 4.3. AACMM-LTS extrinsic calibration

Once the sensor calibration from the captured image has been done, not only the laser line points but also the calibration object points coordinates are known in the LTS global frame. This calibration defines the sensor global reference system that matches the gauge object local coordinate system for the position of image capture. In this way, the matrix that relates the sensor global coordinate system to the AACMM global coordinate system for the AACMM capture image position is the transformation matrix obtained by contact measurement of the gauge object in Equation (15). Rewriting this equation, the expression of Equation (22) is obtained, valid only for the AACMM and LTS position and orientation used in the image capture:

(22) $${\left[\begin{array}{c} x_{i}\\ y_{i}\\ z_{i}\\ 1 \end{array}\right]}_{0\_\mathrm{AACMM}}={}^{\mathrm{AACMM}}M_{\mathrm{W\_LTS}}{\left[\begin{array}{c} x_{i}\\ y_{i}\\ z_{i}\\ 1 \end{array}\right]}_{\mathrm{W\_LTS}}$$

where *^AACMM^M_W_LTS_* = *^AACMM^M_CAL_*.

With Equation (22), laser line points can now be obtained in the AACMM global frame for the calibration position. Once mounted in the arm, obtaining the LTS extrinsic parameters requires the calculation of a new transformation matrix, necessary to express these points in the last AACMM joint frame. The matrix that makes this link is ^6_*AACMM*^*M*_0_*AACMM*_ that will coincide with the inverse matrix of the product of matrices *A_1_* to *A_6_* of Equation (3) corresponding to the AACMM position during calibration image capture. Thereby, it is possible to calculate the desired homogeneous transformation matrix, which will obtain laser line point coordinates related to the last AACMM joint frame for the calibration position:

(23) $${\left[\begin{array}{c} x_{i}\\ y_{i}\\ z_{i}\\ 1 \end{array}\right]}_{6\_\mathrm{AACMM}}={\left({}^{0}T_{6}\right)}_{\mathrm{Calibration\_pos}}^{-1}\cdot {\left({}^{\mathrm{AACMM}}M_{\mathrm{W\_LTS}}\right)}_{\mathrm{Calibration\_pos}}{\left[\begin{array}{c} x_{i}\\ y_{i}\\ z_{i}\\ 1 \end{array}\right]}_{\mathrm{W\_LTS}}$$

With this, it is possible to define the desired matrix by means of Equation (24). This matrix contains the sensor extrinsic parameters that determine the position and orientation of the sensor global coordinate system with respect to the last coordinate system of the kinematic chain of the arm. The terms *pr* form a 3 × 3 rotation matrix and the terms *pt* the translation vector between both coordinate systems:

(24) $$M_{\mathrm{LTS\_Probe}}={\left({}^{0}T_{6}\right)}_{\mathrm{Calibration\_pos}}^{-1}\cdot {\left({}^{\mathrm{AACMM}}M_{\mathrm{W\_LTS}}\right)}_{\mathrm{Calibration\_pos}}=\left[\begin{array}{cccc} {\mathrm{pr}}_{11} & {\mathrm{pr}}_{12} & {\mathrm{pr}}_{13} & {\mathrm{pt}}_{x}\\ {\mathrm{pr}}_{21} & {\mathrm{pr}}_{22} & {\mathrm{pr}}_{23} & {\mathrm{pt}}_{y}\\ {\mathrm{pr}}_{31} & {\mathrm{pr}}_{32} & {\mathrm{pr}}_{33} & {\mathrm{pt}}_{z}\\ 0 & 0 & 0 & 1 \end{array}\right]$$

**Figure 9.**
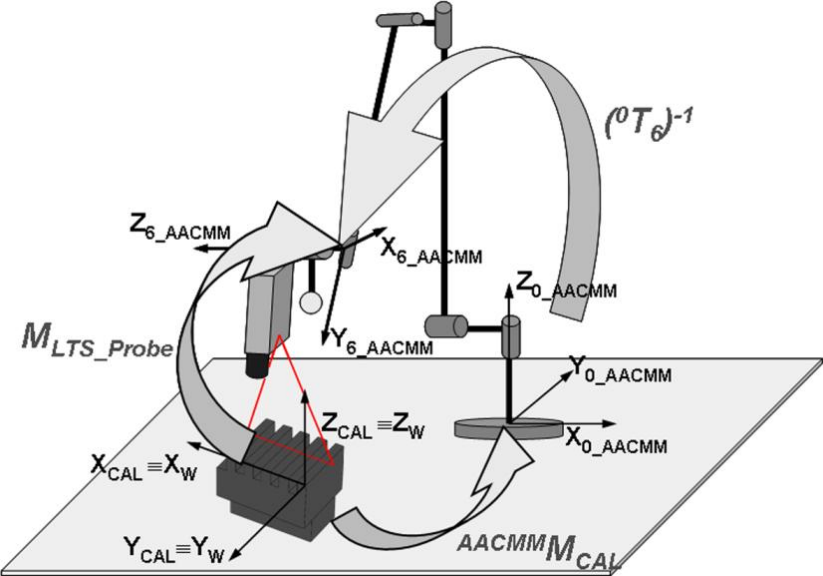
Coordinate systems and transformations in calibration pose.

In Equation (24), a transformation matrix between the LTS and the last AACMM joint frame independent of the AACMM position is obtained, since both systems have coincident movements and both matrices are known. If the position of the sensor according to the calibration object remains constant, this matrix will be the same independently of the AACMM position at the moment of calibration image capture, with small variations occurring due to the error introduced by the AACMM kinematic model geometric parameters. Finally, it will be necessary to apply the AACMM model with the current position *j* geometric parameter values to obtain the captured laser line coordinates in any AACMM position, as shown in Equation (25):

(25) $${\left[\begin{array}{c} x_{i}\\ y_{i}\\ z_{i}\\ 1 \end{array}\right]}_{0\_\mathrm{AACMM}}={\left({}^{0}T_{6}\right)}_{j}\cdot M_{\mathrm{LTS\_Probe}}{\left[\begin{array}{c} x_{i}\\ y_{i}\\ z_{i}\\ 1 \end{array}\right]}_{\mathrm{W\_LTS}}$$

where the vector ${\left[\begin{array}{cccc} x_{i} & y_{i} & z_{i} & 1 \end{array}\right]}_{\mathrm{W\_LTS}}^{T}={\left[\begin{array}{cccc} X_{\mathrm{Wi}} & Y_{\mathrm{Wi}} & Z_{\mathrm{Wi}} & 1 \end{array}\right]}^{T}$ is calculated in Equation (21) for each identified point of the laser line in the image. Matrix *M_LTS_Probe_*, obtained in Equation (24), has been called the “probe matrix”, since integration of both mathematical models produces one more link in the AACMM kinematic chain, replacing the contact probe sphere centre by laser line points related to the LTS global reference frame. Figure 9 illustrates the third step of the process showing the frames and transformations involved. Thus, considering the contact measurement of the gauge object and the capture of one image in a single AACMM position, the LTS intrinsic parameters are obtained; and also the extrinsic parameters that link the LTS global frame and the last joint frame of the AACMM kinematic model in a single step.

## 5. Tests and Results

In order to analyze the accuracy and repeatability of the developed calibration procedure, several calibration tests have been carried out using the FARO AACMM already described. A commercial LTS (DATAPIXEL Optiscan H-1040-L) was linked to the arm. The nominal working characteristics of this sensor are frame rate, 60 fps; working distance, 100 mm; measurement range, 40 mm; field of view, 40 mm; triangulation angle 30°, as well as accuracy, according to the manufacturer, of ±0.010 mm. It is equipped with a 1/3 CCD sensor. This LTS is able to obtain 30,000 pts/sec with nominal repeatability of 10 μm. Previous studies on this sensor mounted in a CMM [2] showed that, in optimal capture conditions, it is able to obtain this level of repeatability in the nominal range, with accuracies of around 100 μm measuring gauge planes and spheres.

Ten different calibrations have been carried out giving 10 *M_LTS_Probe_* matrices in different CMA orientations, the position of the calibration object remaining fixed. The results do not show definitive values of the calibration process repeatability, since important variations in matrix terms are observed, mainly in the translation terms, due to the impossibility of manually fixing the relative position between the LTS and the calibration object. Due to the fact that the LTS reference frame is fixed in the calibration object local frame during its intrinsic calibration, differences between calibrations are expected, since the relative position between this reference system and the last AACMM joint frame changes for each calibration. Therefore, to analyze the calibration influence over the accuracy and repeatability of the captured points, it is necessary to define a method that allows reconstructing the same captured points with the 10 different calibrations made. For this reason, a parametric algorithm has been developed to reconstruct points in the AACMM coordinates from the known *u*, *v* screen coordinates of the captured laser line points, the laser plane equation and the perspective transformation matrix obtained in each intrinsic calibration.

As a repeatability analysis of the calibration procedure, a gauge plane has been digitized obtaining 10 different point clouds for the same plane. For each one of these clouds, the *u*, *v* coordinates of the laser line points have been stored, in addition to the AACMM joint reference frames position for each captured line, so that the laser line points expressed in the AACMM global reference frame can be reconstructed in accordance with the chosen calibration. Therefore, once the clouds are reconstructed, 10 clouds of points for each calibration are obtained. Thus, a total of 100 clouds are calculated, with information of one particular cloud of points reconstructed with 10 different calibrations. Since the aforementioned studies demonstrate the high repeatability of the intrinsic calibration procedure, the results obtained for the digitized plane should show the influence of the extrinsic calibration in the final result.

**Figure 10.**
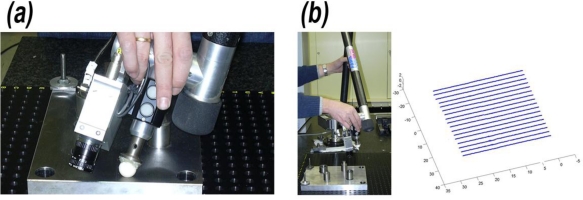
Gauge plane: (a) Contact measurement for nominal data on AACMM global frame. (b) Digitalization.

A plane has been chosen as a gauge geometric primitive in the first test. The nominal value for the plane equation was obtained as the average result from 10 AACMM contact measurements of the plane, Figure 10(a), with 10 points each one, to absorb, as far as possible, the errors derived from the AACMM kinematic model parameters. After that, 10 clouds of points were obtained digitalizing the plane, storing the described parameters for their later reconstruction in AACMM global coordinates as shown in Figure 10(b).

Once the point clouds were reconstructed, the digitized plane equation for each one was determined by a least squares estimation algorithm that includes segmentation and point filtration techniques based on the standard deviation of point distances to the calculated plane. Two error parameters were chosen. Firstly, the angle between the nominal and the calculated plane normal vectors, and finally the difference in the Z coordinate of the central point of the cloud projected on the nominal and the calculated plane. Equations (26) and (27) show the error expressions for cloud *i* corresponding to calibration *j* for the normalized equation values of nominal and measured planes:

(26) $$\varepsilon a_{\mathrm{ij}}=a\;\text{cos}\;\left(A_{N}\cdot A_{\mathrm{ij}}+B_{N}\cdot B_{\mathrm{ij}}+C_{N}\cdot C_{\mathrm{ij}}\right)$$

(27) $${\varepsilon z}_{\mathrm{ij}}=Z_{N}-Z_{\mathrm{ij}}$$

(28) $$\mathrm{Zij}=\frac{-A_{\mathrm{ij}}\cdot X_{C}-B_{\mathrm{ij}}\cdot Y_{C}-D_{\mathrm{ij}}}{C_{\mathrm{ij}}}$$

**Figure 11.**
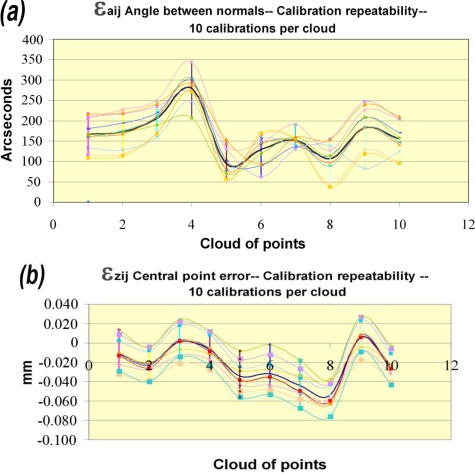
System repeatability and calibration process influence: (a) Angle between normals. (b) Projected central point error.

Figure 11(a) shows the results obtained for the angle between normals. It is possible to see two effects in this figure. Firstly, the error obtained for 10 clouds with each calibration (curves along x axis) is represented. Each one of these curves represents the repeatability of the process of points capture with a single calibration, which is why the variability and the error in this case are referable to the measurement system. The maximum value for all calibrations obtained for this value of system repeatability is around 250 arcsecs, attributable to the repeatability of the AACMM-LTS system for a certain calibration. Secondly, for each cloud of points, the range of the values obtained with each calibration is represented, showing a mean range for the angle between normals due to the calibration procedure of around 100 arcsecs. Thus, a certain influence of the calibration process is observed in the results, although at this point it is not possible to isolate this influence from other error sources due to the process of the capture itself, for example the lack of squareness of the laser plane with respect to the digitized surface, the variations in capturing distance during the digitalization, or the AACMM repeatability.

**Figure 12.**
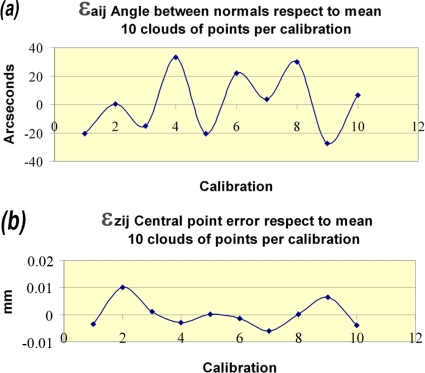
Calibration influence on system repeatability: (a) Angle between normals mean repeatability. (b) Projected central point error mean repeatability.

Figure 12 is aimed at trying to isolate the influence of the calibration process. In the case of Figure 12(a), the mean range of the error for 10 point clouds for each calibration has been calculated obtaining, as mentioned above, values of around 100 arcsecs for each one with low standard deviations. If the variations produced in this range when reconstructing the clouds with different calibrations are calculated, a variation of the average repeatability values within the range of ±30 arcsecs around the mean repeatability value of the system is observed, directly attributable to the calibration process.

Figure 11(b) represents the effect of the calibration process on the Z error. As in the case of the angle between normal vectors, repeatability values for Z within the same calibration of around 60 μm are observed and are mainly attributable to the system repeatability. On the other hand, the calibration influence is appraised again in the system accuracy, introducing variations of 50 μm in the Z error. Figure 12(b) shows the influence of the calibration process on the average system repeatability, introducing maximum variations within a range of ±10 μm.

**Figure 13.**
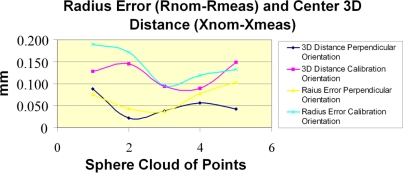
Accuracy estimation of the whole system. Radius and centre distance errors digitizing a reference sphere.

After the repeatability was analyzed, an estimation of the complete system accuracy was made by means of a reference ceramic sphere digitized five times and reconstructing the clouds of points with the calibration close to the average values of error obtained in the angle between the normals and Z. To emphasize the influence of the points capture strategy, the sphere was digitized five times orienting the laser plane perpendicular to the surface, and another 5 times with an orientation of the LTS similar to that used for its calibration. The results of Figure 13 show appreciable differences in the accuracy of the system based on the direction of the laser plane.

The best results are obtained, for orientation of the laser perpendicular to the sphere surface, showing accuracies around of 50 μm both in radius error (R_NOMINAL_-R_MEASURED_) and in distance between centres error. In Figure 13 it must be considered that a ceramic sphere has been used as a reference object, being partially translucent and producing laser penetration into its surface, resulting in a measured radius smaller than the nominal one. The influence of the capture conditions is significant, mainly considering the difficulty of keeping the capture conditions constant in a manually operated measurement system.

## 6. Conclusions

This paper presents an intrinsic and extrinsic LTS-AACMM calibration method, the calibration procedure being performed in a single step with the LTS already mounted in the AACMM, with no need to previously characterize the LTS-Contact probe set geometry by means of calibration methods on CMM. The developed method also avoids the use of approximated techniques to optimize the LTS position and orientation subsequent to the assembly of the sensor in the arm; techniques that are based on contact measurement and digitalization error of gauge primitives in several trajectories. These approximated techniques use estimated initial values of sensor position and orientation and are common practice in almost all commercial AACMM-LTS systems. This achieves a simple and cheap calibration method for the final user, required for any portable measurement equipment. By means of the use of a gauge object that materializes points in different planes with respect to a local reference frame, it is possible to obtain the equation of the sensor laser plane, its perspective transformation matrix and the necessary conjugated pairs of points in the LTS frame and the AACMM frame for the extrinsic calibration in a single operation in any AACMM image capture posture. The experimental results show the repeatability of the calibration process by means of digitalization of gauge primitives, with suitable accuracies for AACMM-LTS digitalization systems.

A procedure of kinematic calibration for AACMMs has also been presented. This method is based on the continuous capture of arm positions by directly probing the centre of the spheres of a gauge ball bar by way of a self-centring kinematic coupling probe. Oppositely, current methods are based on the capture of identification data probing surface points of geometrical primitives of different gauge objects. Parameter identification relies on a Levenberg-Marquardt scheme with an objective function including terms of error in distances and terms of standard deviation which allow to consider the influence of arm repeatability, given its capacity to probe the same point from different orientations.

## Figures and Tables

**Table 1.** Identified values for the model parameters by L-M algorithm [14].

**Joint**	*a_i_***(mm)**	*α_i_***(^o^)**	*d_i_***(mm)**	*θ*_0*i*_**(^o^)**
1	0.036962	−90.052249	−0.000002	−0.126434
2	0.102485	90.044751	47.891183	14.942165
3	0.097868	−90.020699	645.780523	−88.99688
4	−0.133079	90.068899	54.240741	−3.636896
5	0.057606	90.011014	615.242600	89.770488
6	0.367275	−0.522698	0.150712	−0.878373

	*X_probe_* (mm)	*Y_probe_* (mm)	*Z_probe_* (mm)	
	0.367276	139.450887	54.657060	

**Table 2.** Quality indicators for the identified set of model parameters over seven ball bar locations (10780 AACMM positions) [14].

**Distance Error (mm)**		**2σ by Sphere (mm)**	
**Max.**	**0.144258**	**Max.**	**0.249325**
Causing Pos.	POS2	Causing Pos.	POS 1
Causing Dist.	D1	Causing Sph.	B1
Min.	0.005550	Causing Coord.	Z
Causing Pos.	POS1	Min.	0.035286
Causing Dist.	D2	Causing Pos.	POS4
**Med.**	**0.066202**	Causing Sph.	B6
		Causing Coord.	Y
		**Med.**	**0.104355**
